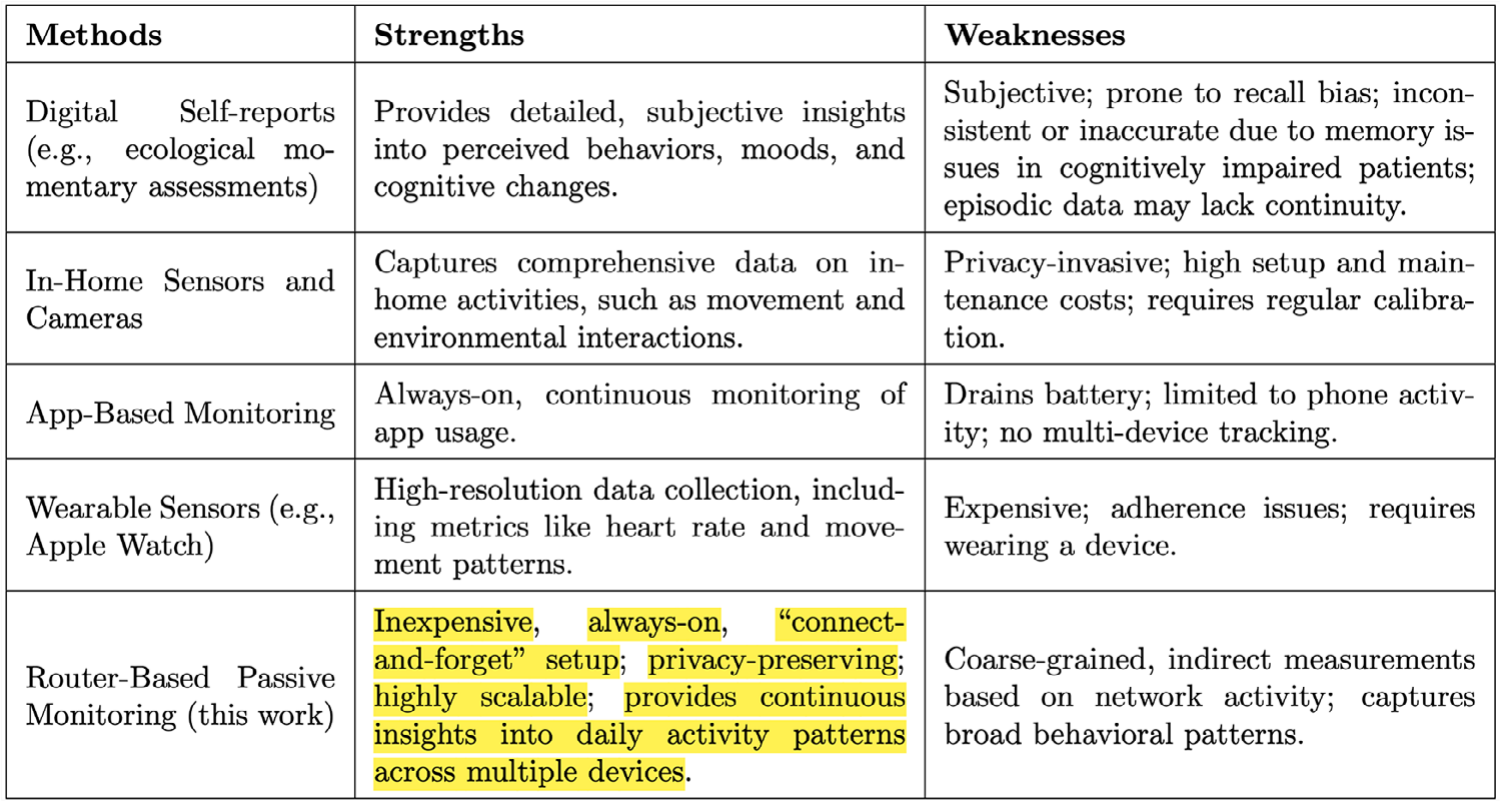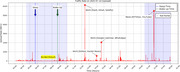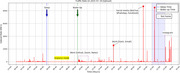# RouterSense: A Passive, Network‐Based Health Monitoring System for In‐Home Patients

**DOI:** 10.1002/alz70863_110660

**Published:** 2025-12-23

**Authors:** Rameen Mahmood, Danny Yuxing Huang

**Affiliations:** ^1^ New York University, Brooklyn, NY USA

## Abstract

**Background:**

Digital biomarkers—sleep disruptions, reduced out‐of‐home activity, and changes in online engagement—serve as early indicators of ADRD, which is characterized by a prolonged preclinical phase. Detecting these subtle changes early can enable timely intervention and slow disease progression. As shown in Table 1, existing monitoring solutions are costly, intrusive, and require user compliance, making them impractical for long‐term use. Traditional assessments, though valuable, are episodic and may overlook early‐stage changes. These challenges underscore the need for a scalable, passive solution for continuous monitoring and early ADRD detection.

**Method:**

RouterSense is a software‐only, plug‐and‐play tool that leverages existing commodity hardware—requiring no additional sensors or specialized hardware. It transforms connected home devices into ambient health sensors by continuously analyzing encrypted network activity to establish baseline behavioral patterns. Using machine learning, it detects deviations in biomarkers such as sleep‐wake cycles, app/device interactions, and time spent at home vs. outside—all biomarkers strongly correlated with ADRD. A preliminary study (*n* = 3) was conducted over a 72‐hour period with cognitively intact participants to evaluate RouterSense's ability to track these behaviors. The participants’ device activity was monitored and compared against self‐reported behavioral logs.

**Result:**

RouterSense successfully identified key digital biomarkers associated with ADRD. It detected nighttime awakenings, as shown in Figure 1, where a participant opened Instagram during sleep hours, indicating disrupted sleep—a common ADRD marker. Figure 2 illustrates shifts in app engagement, with work‐related usage (e.g., Slack, Gmail) during the day transitioning to entertainment (e.g., YouTube) in the evening. Additionally, based on network changes, RouterSense inferred out‐of‐home activity—a behavior that is often impacted in ADRD patients.

**Conclusion:**

RouterSense offers a scalable solution for early ADRD detection through passive network traffic analysis. By continuously analyzing daily behavioral patterns without requiring active user engagement or additional hardware, RouterSense overcomes key limitations of existing monitoring solutions. Future work will expand on these findings through larger, longitudinal studies involving ADRD populations, integrating real‐time alerts for caregivers and clinicians. RouterSense has the potential to transform remote patient monitoring by offering an unobtrusive, cost‐effective method for identifying early ADRD detection, ultimately facilitating timely interventions and improving patient care.